# Mystical and mythological believes not only limited to psychiatric diseases? A dynamic overview of medicine

**DOI:** 10.1097/MS9.0000000000000108

**Published:** 2023-02-17

**Authors:** Anusha Sumbal, Ramish Sumbal

**Affiliations:** Dow University of Health Sciences, Karachi, Pakistan

**Keywords:** Myths, Vampirism, Psychiatric diseases, Mystical belief

## Abstract

The concept of supernatural forces has always been part of medicine and allied science. These beliefs play a pivotal role in the patient-healthcare bond and disease awareness. Traditionally it was believed that psychiatric illnesses are mostly linked with mythologies and paranormal beings since most mental diseases appear to be lunatic and have no rational ground. Paradoxical to this conventional belief we discovered that mythological beliefs have penetrated all fields of medicine. Porphyria, hepatomegaly presenting with photosensitivity, are associated with “vampirism.” Similarly, holoprosencephaly, a congenital anomaly presenting facial deformity is thought to be cyclops folktales. Epilepsy though is simply a neurologic illness believed to be “demonic possession.” Patients of pellagra, a deficiency of vitamin B3 are thought to be werewolves. Thus, we found the presence of mythological association in all types of illnesses. We expect our healthcare infrastructure to not limit their management to counseling patients suffering from psychiatric illnesses only.

The presence of mystical ideology and belief in supernatural powers has always been part of our world. A profound impact of paranormal beliefs and cultural values exists in medicines and allied sciences^1^. These beliefs directly impact patient-healthcare trust and compliance, thereby also controlling speedy recovery and the holistic well-being of patients suffering from various illnesses^2^. An overlook of literature gives us sound knowledge about the presence of mystical beliefs from the ancient era^1^,^2^. Greek myths speak about Apollo, the God of medicine, and believe it to be controlling healing and plaque^3^. Roman folk tales mention Goddess Bona Dea, responsible for controlling fertility and virginity^4^. Metaphysical ideologies point toward unclean spirits, evil possession, and black magical forces^2^. Even in modern times, movies such as *The Exorcist* reflects that these mystical beliefs are deeply engraved in our subconscious^2^. People in our society easily associate the occurrence of any disease with evil spirits and paranormal forces causing the illness^2^.

Unfortunately, the most common illness misinterpreted with paranormal activities is known to be psychiatric disorders^2^. Owing to the fact that most mental diseases appear to be lunatic and have no rationale ground^2^. Almost one-third of people around such patients perceive it to be a paranormal incident, and around 73% of families approach faith healers^5^. Paradoxical to the conventional belief that only mental illness accounts be stigmatized with paranormal belief, it was astonishing to find that the threat not only limits itself to psychiatry but that mystical beliefs have damaged almost all other branches of medicine which were never highlighted^6^–^9^.

We reviewed the literature with a multidisciplinary approach and found that the largely accepted ideology suggesting psychiatric illness is associated with myth is wrong. The problem does not lie like illness; rather, it is such a strong prevalence of myths and the metaphysical world that makes the delusion worse^2^. Also, this metaphysical belief is not limited to any particular tradition or culture, but literature shows that a common perception of healing powers, evil spirits, or supernatural creatures causing illness or recovery in health exit^2^. Porphyria, which is a liver disease presenting itself as hepatomegaly, photosensitivity, and jaundice, has been associated with ‘vampirism^8^.’ Such patients are entitled to be vampires, a dead human who has returned in the form of an evil supernatural creature to kill and suck blood from humans^8^. Holoprosencephaly, which accounts to be 50.3% of termination of pregnancy reported in birth defect clearance system^10^. It is a congenital deformity in which normal separation of the developing fetus forebrain fails, resulting in hypotelorism of eyes with upturned nostrils and lips^6^. Such babies are believed to be Cyclopes, strong one-eyed gigantic supernatural creatures who helped Zeus in Greek folktales^6^. Epilepsy belongs to the neurologic spectrum of diseases; patients with this disorder present seizures due to abnormal electrical rhythm in the brain electrical waves^9^. Unfortunately, even this neurologic pathology is linked to being possessed and descending the evil soul into one’s body^9^. Pellagra, which is just a nutritional deficiency of vitamin B3, is linked to being supernatural creatures, as patients to suffer from erythematous glossitis, which is believed to be fangs with dripping blood, pruritus, insomnia, and rage, which is a classic description of werewolves mentioned in vampire folktales^7^,^8^.

Thus, unlike the traditional belief that only psychiatric diseases are labeled as paranormal diseases, we found that mystical beliefs are not limited to mental illness^1^. Rather regardless of the etiology of the disease, people easily join it with supernatural beings and mythological folktales^2^. Literature has never addressed such a dynamic involvement of occult powers in other metabolic, systemic, and congenital diseases. We believe that such fallacies are very debilitating for such patients^11^. Families of these patients, and sometimes the patient themselves, never realize that these are merely medical conditions, not a folktale or curse, and thus they never show up to any doctor or healthcare provider^11^. These patients are deprived of the basic right to treatment and are victims of mystical beliefs^11^. This individual suffers from the disease itself and stigmatization by society, which could be cured in the very first place^11^.

We expect our healthcare infrastructure to comprehend the point mentioned here and do not limit their management in counseling of patients and family members suffering from psychiatric illnesses only but to take into account all other diseases as well. Duty resides on the doctor and paramedics this time to understand this dynamic overview and pave the way to holistic management in our perspective of health and allied sciences.

## Ethical approval

Not applicable.

## Patient consent

Not applicable.

## Sources of funding

None to declare.

## Author contribution

A.S. and R.S: concept and writing the paper.

## Conflicts of interest disclosure

There are no conflicts of interest.

## Research registration unique identifying number (UIN)

1. Name of the registry: not applicable.

2. Unique identifying number or registration ID: not applicable.

3. Hyperlink to your specific registration (must be publicly accessible and will be checked): not applicable.

## Guarantor

None to declare.

## Provenance and peer review

Not commissioned, externally peer-reviewed.
